# Bibliometric Insights Into the Evolving Landscape of Antibiotic Resistance Research: Trends, Collaborations, and Key Foci (1965-2023)

**DOI:** 10.7759/cureus.53508

**Published:** 2024-02-03

**Authors:** Namrata Dagli, Mainul Haque, Santosh Kumar

**Affiliations:** 1 Dentistry, Karnavati Scientific Research Center (KSRC), Karnavati School of Dentistry, Karnavati University, Gandhinagar, IND; 2 Pharmacology and Therapeutics, National Defence University of Malaysia, Kuala Lumpur, MYS; 3 Periodontology and Implantology, Karnavati School of Dentistry, Karnavati University, Gandhinagar, IND

**Keywords:** antibiotic resistance, antibiotic resistance research, key aspect, partnerships, movement, understandings, network visualization, network analysis, bibliometric

## Abstract

Conducted on randomized clinical trials (RCTs) addressing antibiotic resistance in the PubMed database, this bibliometric analysis explores relevant sources, keyword co-occurrence, institutional co-authorship, global collaboration patterns, and evolving research trends. Utilizing an electronic search on January 13, 2024, employing the term "antibiotic resistance," 252,657 results were retrieved, of which 2,962 RCTs were analyzed. The dissemination of RCTs exhibited a variable distribution from 1965 to 2023, with a peak in 2014, noteworthy peaks in 1993-1994 and 2002-2003, contrasting declines in 1990-1991 and 2007-2008, and a consistent decrease post 2018. The University of California emerged as a predominant institution, and the journal "Antimicrobial Agents and Chemotherapy" substantially contributed. The annual growth rate stood at 1.2%, with 97 single-authored documents, an average of 8.76 co-authors per document, and 8.89% international co-authorships. Co-occurrence analysis highlighted prevalent themes, including double-blind clinical trials and significant keywords like human immunodeficiency virus (HIV) infections, *Helicobacter* infections, metronidazole, and amoxicillin. Trend analysis revealed a chronological shift from penicillin to HIV and *Helicobacter* drug therapies, culminating in combination antibacterial therapy for multiple bacterial strains. The prevailing trend in antibiotic resistance publications involved single-country endeavors, with the United States leading in collaboration frequency. The findings indicate a need to foster international collaboration, promote interdisciplinary research, support emerging trends, encourage open-access publication, and address declines in research activity, particularly RCTs.

## Introduction and background

In an era of unprecedented medical advancements, antibiotic resistance is a formidable challenge, threatening to compromise the efficacy of our most potent tools in combating infectious diseases. A recent study reported that approximately 1.27 million deaths were directly attributable to bacterial antimicrobial resistance in 2019. A diverse set of pathogens is involved, and resistance is high for many other drugs, including β-lactams (e.g., penicillin and methicillin) and fluoroquinolones (e.g., ciprofloxacin) [1]. The escalation of antibiotic resistance poses a global threat, as outlined in the 2022 Global Antimicrobial Resistance and Use Surveillance System (GLASS) report, which reveals alarming resistance rates among prevalent bacterial pathogens. The report highlights concerning median rates, such as 42% for third-generation cephalosporin-resistant* Escherichia coli *and 35% for methicillin-resistant *Staphylococcus aureus* (MRSA) across 76 countries. Notably, urinary tract infections caused by *E. coli* exhibited reduced susceptibility to standard antibiotics in 2020, making effective treatment more challenging. *Klebsiella pneumoniae* displayed elevated resistance levels, potentially leading to increased dependence on last-resort drugs like carbapenems. Projections indicate a twofold surge in resistance to last-resort antibiotics by 2035. Drug resistance is also observed in human immunodeficiency virus (HIV), tuberculosis, malaria, and neglected tropical diseases, necessitating vigilant surveillance, strategic interventions, and strengthened global collaboration to address this escalating public health crisis [2,3]. Antibiotic resistance poses a multifaceted threat, impacting public health, agriculture, and the environment [4,5]. The growing prevalence of resistant strains and the diminishing pipeline of novel antibiotics underscore the urgency of understanding the global research landscape surrounding this phenomenon [6]. As the worldwide healthcare community grapples with the escalating antibiotic resistance crisis, it becomes imperative to employ rigorous analytical methods to gain deeper insights into the evolving landscape of this pressing issue [7].

A bibliometric analysis offers a panoramic view of the research outputs, collaborations, and citation patterns in the field, shedding light on the interconnected web of scientific contributions that collectively shape our understanding of antibiotic resistance. This article embarks on a bibliometric journey, employing a systematic and quantitative approach to assess the scientific literature on antibiotic resistance. By delving into the rich repository of scholarly publications, we aim to map the intellectual terrain, identify critical contributors, and unearth emerging trends in the ongoing battle against antibiotic resistance. By critically evaluating the existing literature, we aim to provide a foundation for informed decision-making, guiding future research endeavors and policy formulations to mitigate the impact of antibiotic resistance.

## Review

Materials and methods

Search Strategy and Study Selection Process

An online electronic search was performed on January 13, 2024, in the PubMed database to identify the research done on antibiotic resistance. The search term used was antibiotic resistance. No filter was applied in the PubMed database. A total of 252,657 papers appeared in an online search, of which 5,130 were clinical trials. Of these 5,130 papers, 2,926 were randomized clinical trials (RCTs). All RCTs were exported in a text file for the bibliometric analysis. The study selection process flowchart was generated according to the Preferred Reporting Items for Systematic Reviews and Meta-Analyses (PRISMA) guidelines [8].

Data Analysis

Network analysis and visualization were performed using the VOSviewer software, Version 1.6.20 [9], and Biblioshiny, a web-based app for comprehensive science mapping analysis [10]. Biblioshiny analyzed the author's productivity and core sources.

Results

The Search Results for the Research Papers on Antibiotic Resistance

**Figure 1:**
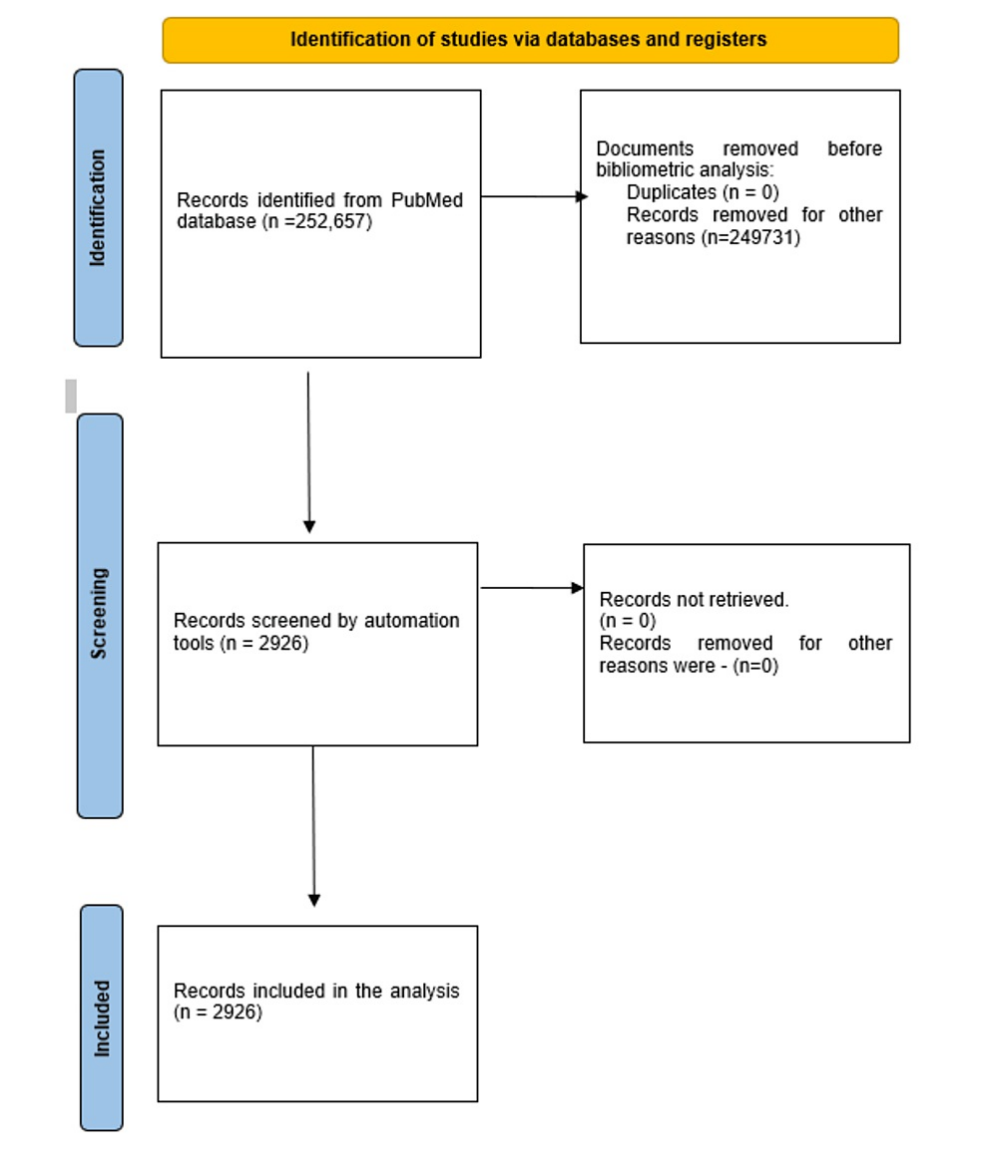
PRISMA flowchart illustrating the process of selecting studies for the analysis PRISMA: Preferred Reporting Items for Systematic Reviews and Meta-Analyses

Upon conducting an online search without applying filters, 252,657 results were obtained. Among these, 5,130 were clinical trials, comprising 2,926 randomized controlled trials, 31,371 were reviews, 1,110 were meta-analyses, and 112 were book chapters and other documents. For the analysis, only RCTs were chosen (Figure 1). The annual growth rate stands at 1.2%. There were 97 single-authored documents. The average number of co-authors per document is 8.76. International co-authorships accounted for 8.89%.

The Publishing Trend of RCTs on Antibiotic Resistance

**Figure 2:**
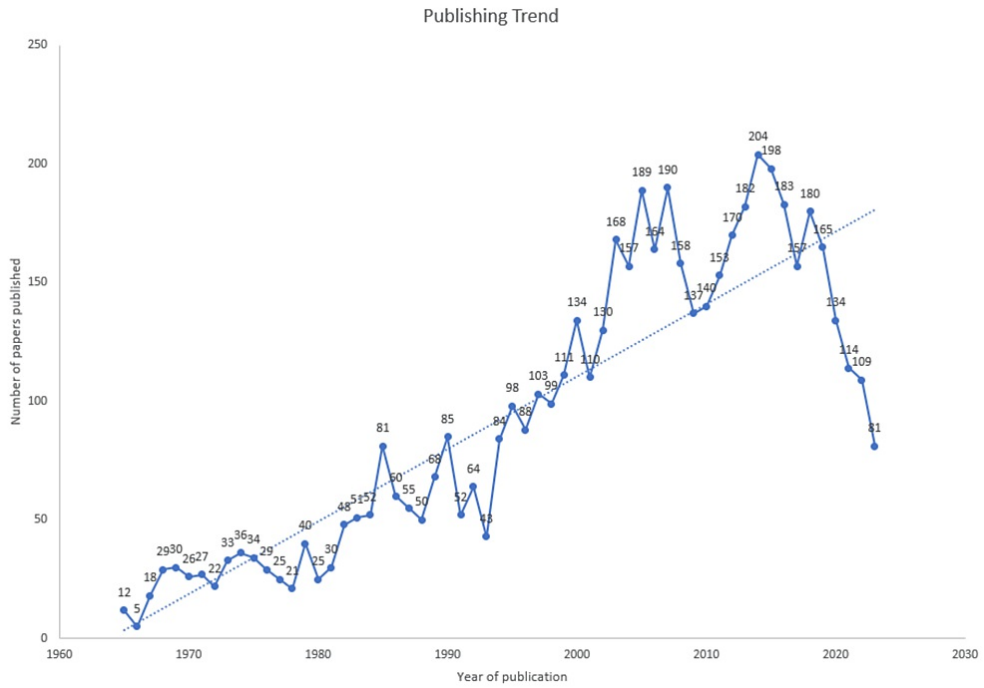
The publishing trend of RCTs on antibiotic resistance RCTs: randomized clinical trials

Figure 2 illustrates an erratic distribution pattern in the publication of RCTs from 1965 to 2023. The depicted trend line reflects a consistent increase over the past 58 years, reaching its pinnacle with the highest number of RCTs published in 2014. The most substantial surge occurred during 1993-1994, with 40 papers, followed closely by 2002-2003, with 38 papers. A notable downturn in the number of published documents is evident in 1990-1991 (33 papers), succeeded by a similar decline in 2007-2008 (32 papers). After 2018, a persistent decrease in published RCTs is observable.

**Figure 3:**
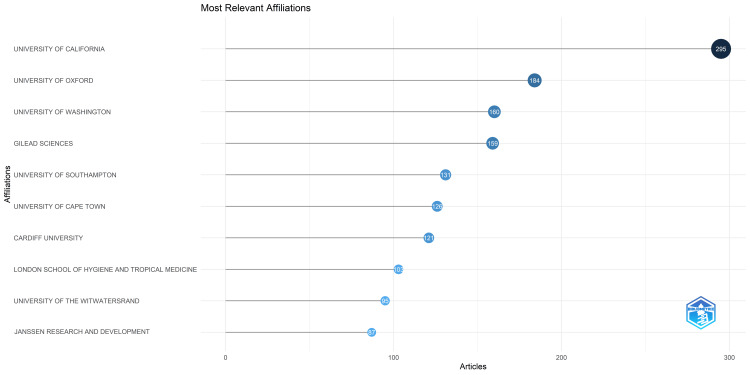
Most relevant institutions in antibiotic resistance research

The preeminent institution in the field is the University of California, which produced 295 papers, followed by the University of Oxford, the University of Washington, and Gilead Sciences, which contributed 184, 160, and 159 research papers, respectively (Figure 3). The top 10 institutions collectively published 1,461 research papers.

Analysis of Key Institutions, Co-authorship Patterns, and Productivity Metrics

**Figure 4:**
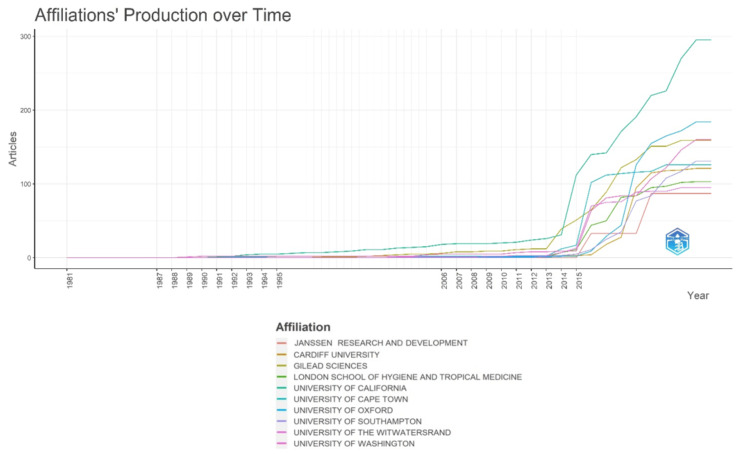
Institution's productivity analysis

Figure 4 illustrates that the initial surge in research papers on antibiotic resistance emerged from Gilead Sciences in 2013, succeeded by the University of California. In other academic institutions, the surge is observed after 2015.

**Figure 5:**
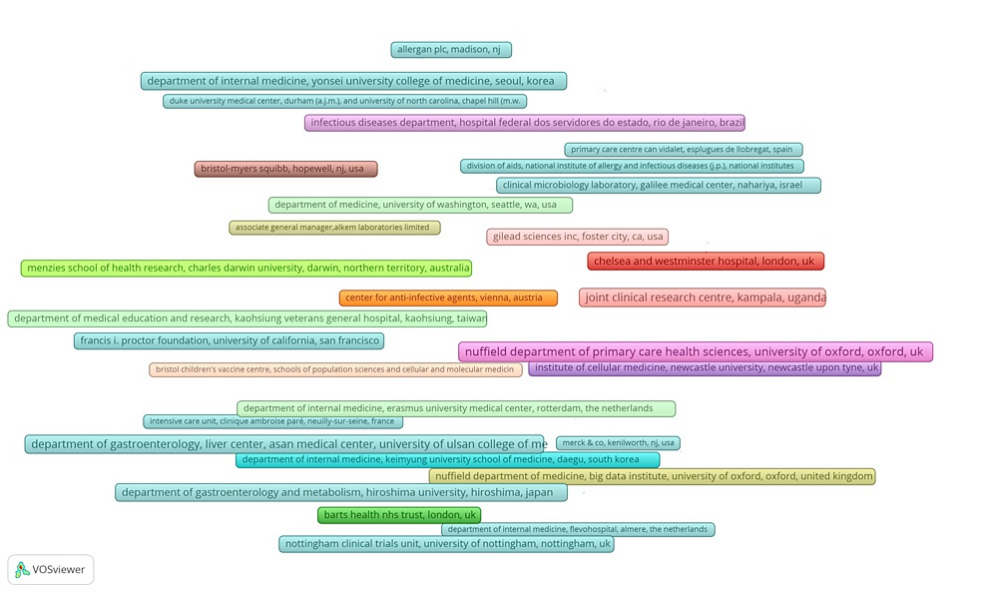
Co-authorship analysis for institutions. Weight: documents. Maximum length of frames: 100

Figure 5 depicts the most relevant sources.

Most Relevant Sources

**Figure 6:**
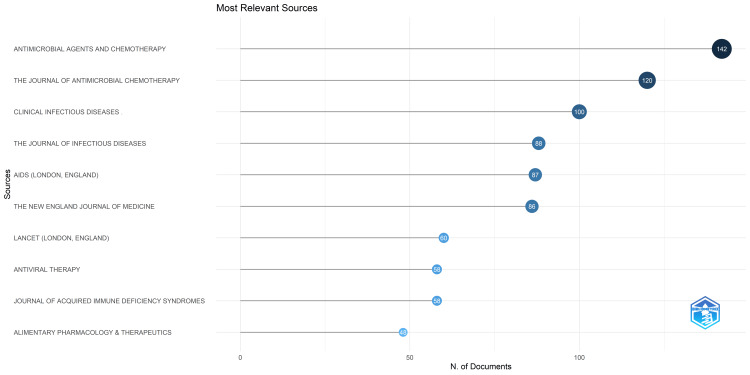
Most relevant sources

**Figure 7:**
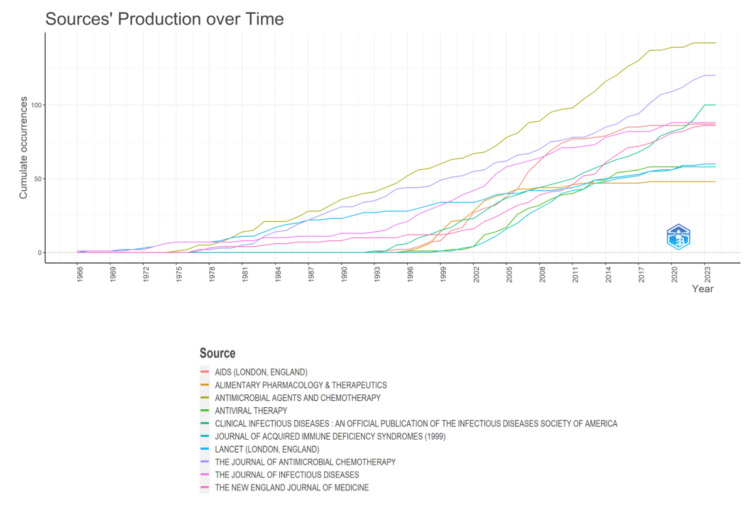
Sources' production over time for the most cited research papers

The preeminent source, "Antimicrobial Agents and Chemotherapy," comprising 142 research papers, accounted for nearly 17% of the publications across the 10 most pertinent journals, amounting to 847 documents. Another noteworthy contributor is the Journal of Antimicrobial Chemotherapy, which presented 120 research papers, constituting 14.16% of the aggregate output (Figure 6). The graphical representation delineates the temporal evolution of journal productivity. Notably, two prominent surges are discernible in the graph: the first occurring around 1975 and the second from 1993 to 2002. The latter surge is characterized by a substantial escalation in the number of papers across all sources (Figure 7).

Keyword Analysis

**Figure 8:**
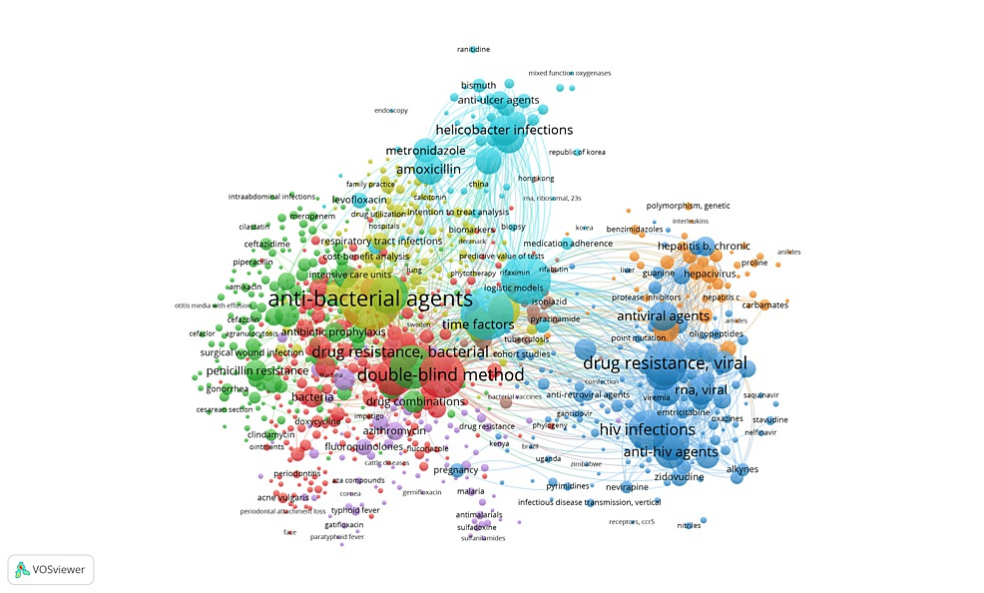
Network analysis of keyword co-occurrence. Weight: occurrences

The analysis of keyword co-occurrence was conducted using the VOSviewer software. The analysis unit employed Medical Subject Headings (MeSH) keywords, and the counting method chosen was full counting. A total of 3,252 MeSH keywords were identified, comprising 1,859 with a minimum occurrence of 2, 1,365 with a minimum occurrence of 3, 1,112 with a minimum occurrence of 4, and 10 with 603 occurrences. We specifically selected keywords with a minimum occurrence of 5, resulting in 965 keywords meeting the criteria. Subsequently, the software calculated the total strength of co-occurrence links for each of the 965 keywords, and those with the highest real link strength were chosen for further analysis. The network analysis revealed eight clusters, with item counts ranging from 31 to 254, 48,643 links, and 174,688 total link strength (Figure 8).

The prevailing keywords, double-anonymized method, antibacterial, and antiviral agents, suggested a prevalent use of double-anonymized clinical trials in investigating antibacterial and antiviral drug resistance. Additional keywords identified through network analysis with high occurrences included HIV infections, *Helicobacter* infections, metronidazole, and amoxicillin. Other drugs that are studied less and/or used less frequently as keywords are penicillin, methicillin, azithromycin, clindamycin, fluoroquinolones, gatifloxacin, sulfonamides, levofloxacin, meropenem, isoniazid, pyrazinamide, etc.

**Figure 9:**
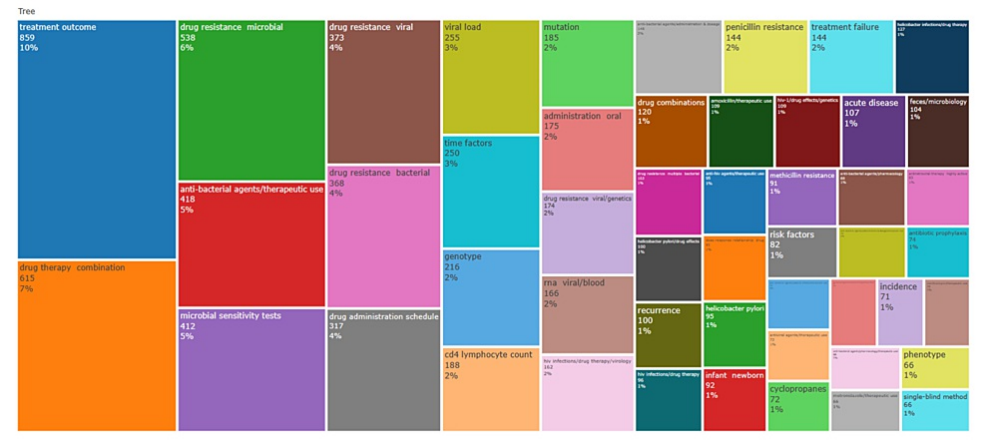
Treemap

A treemap (Figure 9), generated using the Biblioshiny app, corroborated similar results. In creating Figure 8 and Figure 9, keywords not specific to the article's subject were excluded to yield a more detailed and pertinent representation (humans, female, male, adult, middle-aged, young adult, child, preschool, and clinical trial as a topic).

Trend Topic Analysis

**Figure 10:**
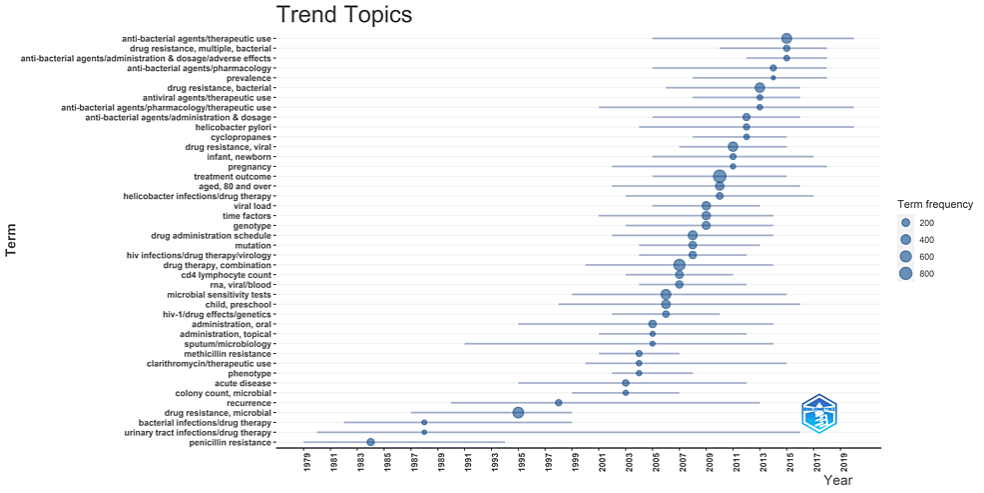
Trend topic analysis

The trend analysis depicted in Figure 10 illustrates a chronological evolution in research focus, initially centered on penicillin, transitioning to HIV drug therapy, and progressing to *Helicobacter* drug therapy, cyclopropane, ultimately culminating in antibacterial therapy for multiple bacterial strains. Prominent terms employed in these investigations include "treatment outcome," "microbial drug resistance," and "combination drug therapy," reflecting prevalent concerns in the field. Notably, the emergence of the less frequently utilized but more recent terminology, "multiple bacterial drug resistance," underscores an escalating severity in the landscape. This observation suggests a growing trend wherein various bacterial species increasingly resist conventional drug therapies, signaling a critical and pressing issue in the domain.

Notably, the emergence of the less frequently utilized but more recent terminology, "multiple bacterial drug resistance," underscores an escalating severity in the landscape. This observation suggests a growing trend wherein various bacterial species increasingly resist conventional drug therapies, signaling a critical and pressing issue in the domain.

Analysis of Author's Countries

**Figure 11:**
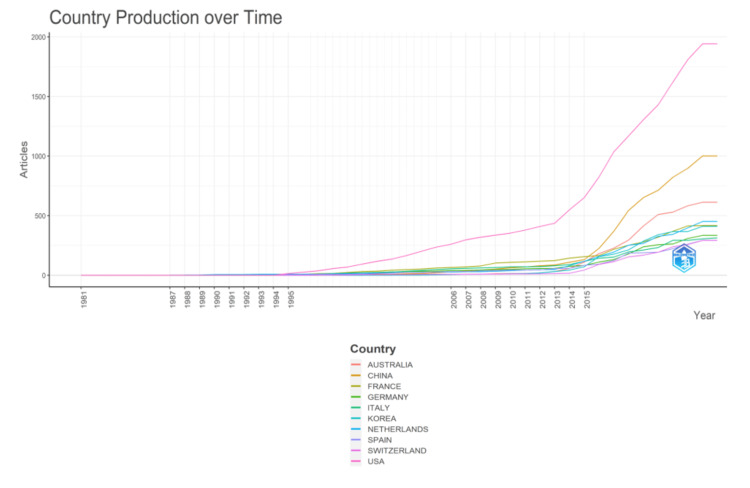
Country production over time

**Figure 12:**
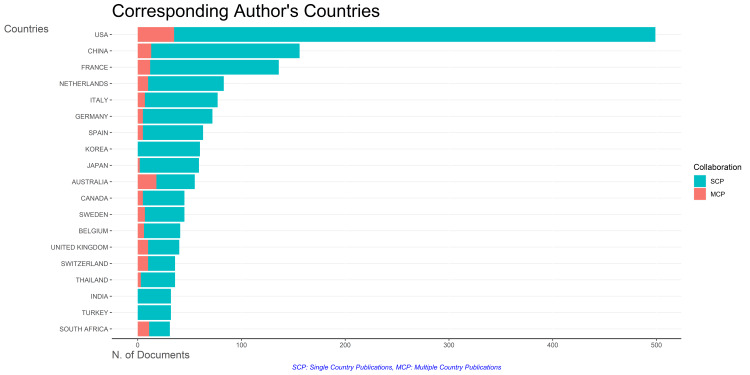
Collaboration of corresponding author's countries

**Figure 13:**
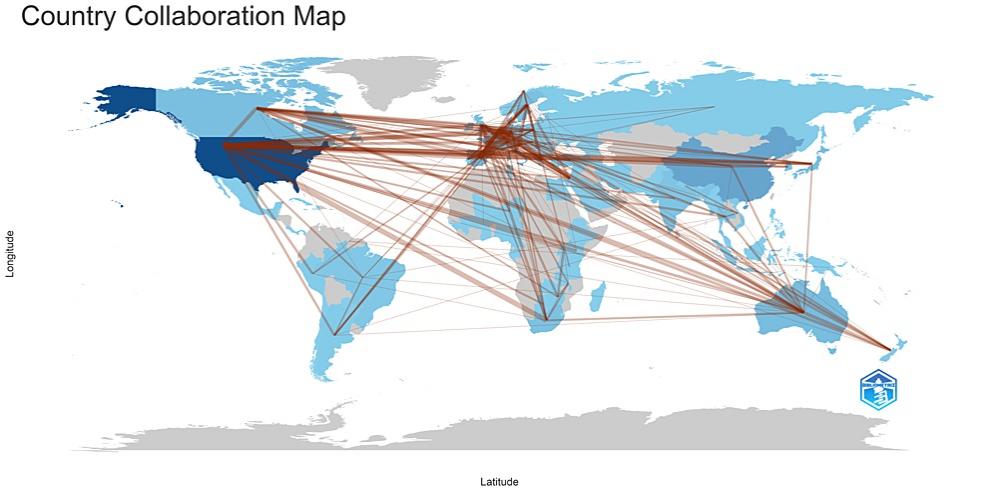
Country collaboration map

Figure 11 illustrates the productivity of nations through a comprehensive analysis of their production trends over time. Figure 12 indicates that most publications addressing antibiotic resistance emanate from individual countries. The United States exhibits the highest frequency of collaborations, followed by Australia. In contrast, countries such as Korea, India, and Turkey do not collaborate with other nations. Figure 13 visually represents the collaborative landscape among these countries in the context of antibiotic resistance research.

Discussion

The analysis of RCTs on antibiotic resistance revealed a fluctuating distribution pattern from 1965 to 2023, experiencing consistent growth over 58 years with notable peaks in 2014, 1993-1994, and 2002-2003, alongside declines in 1990-1991 and 2007-2008 and a persistent decrease post 2018. The possible reason for the decrease could be that events like global pandemics or other public health emergencies might have redirected research efforts and resources towards urgent priorities, impacting the planning and execution of RCTs. Noteworthy contributions came from the University of California, the leading institution, and the "Antimicrobial Agents and Chemotherapy" journal. The annual growth rate is 1.2%, with 97 single-authored documents, an average of 8.76 co-authors per document, and 8.886% international co-authorships. Co-occurrence analysis of keywords identified eight clusters emphasizing double-blind clinical trials, with significant terms including HIV infections, *Helicobacter* infections, metronidazole, and amoxicillin. Trend analysis showcased a chronological progression in research focus, culminating in combination antibacterial therapy. Most antibiotic resistance publications were single-country endeavors, with the United States leading in collaboration frequency. The study utilized the VOSviewer software to conduct a co-occurrence analysis of 3,252 MeSH keywords, identifying 965 with a minimum occurrence of 5, revealing eight clusters in network analysis that highlighted the prevalence of double-blind clinical trials in investigating antibacterial and antiviral drug resistance, with additional noteworthy keywords including HIV infections, *Helicobacter* infections, metronidazole, and amoxicillin. Less studied or less commonly utilized keywords include drugs such as penicillin, methicillin, azithromycin, clindamycin, fluoroquinolones, gatifloxacin, sulfonamides, nevirapine, stavudine, levofloxacin, meropenem, isoniazid, pyrazinamide, etc. The trend analysis reveals a chronological progression in research focus from penicillin to HIV, cyclopropane, and *Helicobacter* drug therapies, culminating in combination antibacterial therapy for multiple bacterial strains.

Our investigation centered on antimicrobial resistance, whereas the primary aspect of HIV treatment involves antiretroviral drugs. Nevertheless, antimicrobials might be separately prescribed to manage bacterial infections in individuals with HIV/acquired immunodeficiency syndrome (AIDS). A thorough examination of the included studies can elucidate the prominence of "HIV drug therapy" as a critical term, which is beyond the scope of this article.

A bibliometric study on antibiotic resistance done by Sun et al. [11] provides a comprehensive overview of the global threat posed by antibiotic-resistant bacteria (ARB). The research highlights the significant contributions of both developed countries, such as the United States, and developing countries, exemplified by China, in addressing ARB in the Web of Science (WOS) database. The interdisciplinary nature of ARB research is underscored, with a primary focus on environmental and microbial aspects. The study emphasizes that antibiotic resistance is a crucial research area and a notable hotspot, despite some progress with novel antibiotics, e.g., lefamulin and cefiderocol [12,13]. A bibliometric analysis of vancomycin concluded that analysis of top-cited articles regarding vancomycin use in children and neonates reveals an equal distribution among infectious diseases and pediatrics journals. Clinical Infectious Diseases emerges as the most productive journal. The study emphasized the need for the data from pediatric population. Key topics include complicated infections and antibiotic resistance/MRSA treatment. Over the past three decades, antibiotic stewardship and new dosing strategies in the pediatric population have gained importance, warranting further exploration [14]. One more study concludes that further research is essential to address the ongoing challenges posed by ARB [11]. Another bibliometric study conducted by Gómez-Ríos and Ramírez-Malule [15] focusing on multidrug and antibiotic resistance articles from 2017 to 2018 highlights research concentration on critical pathogenic bacteria such as *E. coli, Pseudomonas aeruginosa, Acinetobacter baumannii*, and* K. pneumoniae*. The study identifies the United States, China, and India as the most productive countries, with Iran leading in productivity when accounting for gross domestic product [16]. The findings of these studies do not match our study's findings, except for the leading contributor country. Another bibliometric analysis, including 2,611 research articles, reported a notable surge in annual publications from 2011 to 2019. Primary research themes include the dissemination and abundance of antibiotic-resistant genes and the detection of bacterial strains or antibiotic residues in various environmental isolates. Most articles originated from the European region, with China leading in document count and focusing on penicillin/cephalosporin drug classes and *E. coli* as the most frequently encountered pathogen. The Science of the Total Environment journal emerged as the most prolific source [17]. The results of these studies cannot be directly compared with our study due to variations in the databases utilized for data collection and differences in the selected periods.

Our study observations highlight a diminishing trend in the number of studies in the PubMed database addressing this critical issue over the last five years. Nevertheless, since PubMed is among the largest repositories of published scientific literature, further analysis of additional databases is necessary to corroborate this trend.

Several other bibliometric analyses have delved into antibiotic resistance, each focusing on specific topics such as *K. pneumoniae*, pulmonary tuberculosis, carbapenem, the aquatic environment, and wildlife [18-21]. Notably, no other bibliometric analysis on this subject has been conducted on the PubMed database for such an extended period. Investigating the inappropriate utilization of antimicrobials is essential across all nations to establish suitable policies to curb antimicrobial resistance [22].

Limitations of this study

This study analyzed the research papers on antibiotic resistance published in the PubMed database to provide an overview. Through this bibliometric analysis, we aspire to contribute to the ongoing discourse surrounding antibiotic resistance, fostering a deeper understanding of the current state of knowledge while fostering collaboration and innovation in the scientific community. As we navigate the intricate web of publications, citations, and collaborations, we endeavor to uncover insights that will ultimately help safeguard the efficacy of antibiotics for generations to come. The study's main limitation is that only one database was considered for the analysis, as it is not possible to merge the data from two different databases for analysis. Another limitation is that the citation analysis is not included as data from the PubMed database doesn't support such analysis. Moreover, assessing the relevance of each article is impractical due to the large volume of articles. Nevertheless, this study provides a comprehensive analysis of the literature on antibiotic resistance.

Future study recommendation

Future research in antibiotic resistance should focus on exploring the molecular and genetic aspects of resistance evolution, particularly in identifying novel resistance patterns. Additionally, there is a need for in-depth investigations into the efficacy and impact of combination antibacterial therapies, with a specific emphasis on addressing multiple bacterial strains. Understanding the role of the human microbiome in antibiotic resistance and its implications for personalized treatment approaches is crucial. The identified keyword clusters, including HIV infections, *Helicobacter* infections, and specific antibiotics, warrant further investigation to uncover nuanced connections. International collaboration remains essential, and future studies should aim to foster global partnerships to consider regional variations in antibiotic resistance dynamics. Lastly, integrating advanced technologies like artificial intelligence and machine learning in research methodologies could enhance the efficiency and depth of studies, providing innovative solutions to combat antibiotic resistance effectively.

## Conclusions

The publication of RCTs in antibiotic resistance research displayed a fluctuating distribution pattern from 1965 to 2023, peaking in 2014 with notable surges in 1993-1994 and 2002-2003, along with declines in 1990-1991 and 2007-2008 and a persistent decrease post 2018. The University of California emerged as the leading institution, followed by the University of Oxford, the University of Washington, and Gilead Sciences. The journal, "Antimicrobial Agents and Chemotherapy," emerged as the ultimate source, followed by the "Journal of Antimicrobial Chemotherapy." The annual growth rate is 1.2%, with 97 single-authored documents, an average of 8.76 co-authors per document, and 8.886% international co-authorships. Analysis of MeSH keywords highlighted the prevalence of double-blind clinical trials and significant keywords such as HIV infections, *Helicobacter* infections, metronidazole, and amoxicillin. Less commonly utilized keywords include drugs such as penicillin, methicillin, azithromycin, clindamycin, fluoroquinolones, gatifloxacin, sulfonamides, nevirapine, stavudine, levofloxacin, meropenem, isoniazid, and pyrazinamide. The trend analysis revealed a chronological progression in research focus from penicillin to HIV and *Helicobacter *drug therapies, culminating in combination antibacterial therapy for multiple bacterial strains. Most antibiotic resistance publications are single-country endeavors, with the United States leading in collaboration frequency. The decrease in RCTs observed over the past five years is a cause for concern. Additional research is necessary to address this pressing issue comprehensively.
